# Comparison of recombinant human follicle stimulating hormone (rhFSH), human chorionic gonadotropin (HCG) and human menopausal gonadotropin (HMG) on semen parameters after varicocelectomy: a randomized clinical trial

**Published:** 2012-09

**Authors:** Mohammad Ali Amirzargar, Mahnaz Yavangi, Abbass Basiri, Sayyed Mahdi Hosseini Moghaddam, Hooshang Babbolhavaeji, Nasibeh Amirzargar, Hossein Amirzargar, Leila Moadabshoar

**Affiliations:** 1 *Department of Urology, Hamadan University of Medical Sciences, Hamadan, Iran.*; 2 *Department of Obstetrics and Gynecology, Fatemieh Infertility Research Center, Hamadan University of Medical Sciences, Hamadan, Iran.*; 3 *Urology and Nephrology Research Center, Shahid Labbafinejad Medical Center, Shahid Beheshti University of Medical Sciences, Tehran, Iran.*

**Keywords:** *Male Infertility*, *Semen Analyses*, *Varicocele*, *Varicocelectomy*, *HCG*, *HMG*, *rhFSH*

## Abstract

**Background:** The most frequent physical finding in infertile men is varicocele, in which one of the mechanisms that can affect seminal parameters is oxidative stress.

**Objective:** Our study aimed, for the first time, to compare the efficacy of recombinant human follicle-stimulating hormone (rhFSH), human chorionic gonadotropin (HCG) and human menopausal gonadotropin (HMG) on sperm parameters and fertility after varicocelectomy.

**Materials and Methods:** 113 infertile men with varicocele were divided into four groups. Group A received HCG 5000 IU weekly, group B received HMG 75 IU three times a week, group C received rhFSH 75 IU three times a week and group D received no medical treatment after varicocelectomy.

**Results:** After three months, in group A sperm morphology improved (p=0.007), causing a 32% pregnancy rate. In group B, sperm motility (p=0.023) and morphology (p=0.014) improved, causing a 57% pregnancy rate. In group C, all of the investigated semen parameters increased (p<0.05), causing a 62.5% pregnancy rate. Only rhFSH improved sperm concentrations to >20×10^6^ mL (p=0.027). In group D, sperm morphology increased (p=0.038), but other parameters remained unchanged and no pregnancies occurred.

**Conclusion:** It can be concluded that drugs can reduce induction time for spermatogenesis and fertility in comparison with varicocelectomy alone. For these purposes, rhFSH is more effective than other drugs.

## Introduction

Infertility is considered a major public health issue, as it affects about 15% of reproductively- aged couples (1). The male partner is involved in 40-50% of infertility cases with the most frequent physical finding in infertile men being varicocele (2, 3). It has been implicated as a cause in 35-50% of patients with primary infertility and up to 81% of men with secondary infertility (4, 5). Varicocele is associated with testicular volume loss and endocrine abnormalities and this condition can affect seminal parameters, which usually vary from normal to mild or moderate asthenospermia, teratospermia or asthenoteratospermia (6, 7). Varicocele has some adverse effects on spermatogenesis. For instance, if intratesticular pressure increases, attenuation of blood flow causes hypoxia and increased testicular temperatures, also toxic metabolites originated from adrenal glands can reflux, and with the abnormalities of hormonal profile can damage DNA, moreover integration of proteins in the spermatic tubule cells and/or Leydig cells can produce oxidative stress which has recently been shown, surgical varicocelectomy can reduce it in infertile men (8-17). 

Many drugs have been proposed in association with surgery. Gonadotropin therapy has been available for over four decades (18, 19) and has been applied in cases of idiopathic male infertility for stimulation of spermatogenesis. Clinical studies suggest that prolonged HCG administration may initiate (20, 21), maintain (22, 23) or reinitiate (24) spermatogenesis. It has also been shown that empiric use of postoperative HCG in subfertile men who underwent surgical correction of varicocele significantly improved patient results (25). 

Excellent results were obtained after HCG treatment in infertile men who underwent varicocelectomy but did not respond to the operation (26). It has also been recommended that administration of HCG to patients who undergo varicocelectomy, but have persistent subtle Leydig cell dysfunction, may stimulate the intratesticular testosterone production (27). In later studies, significant improvements in sperm parameters and pregnancy rates were observed in patients treated with human menopausal gonadotropin (HMG) with respect to those treated with only varicocelectomy (28). 

Additionally, follicle-stimulating hormone (FSH) treatment in young people with varicocele leads to a statistically significant increase in the seminal fluid parameters (29). In another investigation, it was shown that FSH treatment in patients after varicocelectomy could improve spermatogenesis, particularly in those whose sperm quality was the most compromised prior to treatment (30). 

The objective of our study was to compare the safety and efficacy of recombinant human follicle-stimulating hormone (rhFSH), HCG and HMG on sperm parameters and fertility after varicocelectomy for the first time.

## Materials and methods

This randomized clinical trial was designed as a multicenter, randomized clinical trial. It was conducted in two centers in the Tehran and Hamadan provinces in Iran according to a protocol approved by the local institutional ethical committees of the study centers. Our funding source was Urology and Nephrology Research Center of Shahid Beheshti University of Medical Sciences and Fertility and Infertility Research Center of Hamadan University of Medical Sciences.

One-hundred thirteen infertile patients diagnosed with varicocele who were referred to the Fatemieh Infertility Research Center of Hamadan University of Medical Sciences and the Urology and Nephrology Research Center of Shahid Labbafinejad Medical Center in Tehran and personal offices were enrolled to compare the efficacy and safety of drugs to induce spermatogenesis and fertility after varicocelectomy.

Patients were recruited from December 2008-2009. At the first screening, detailed medical histories of the patient and female partner were taken and a complete physical, hormonal and semen examination was performed. Then participants were eligible for inclusion in the study if they fulfilled the following criteria: male factor infertility due to varicocele diagnosed by an expert urologist, infertility clinically defined as failure to conceive after 12 months of unprotected intercourse during which pregnancy had not been achieved, spontaneous onset of maturation and normal sexual development. Exclusion criteria were as follows: presence of infertility due to any other factor especially female infertility as diagnosed by an expert gynecologist, clinically significant systemic disease, underlying testis abnormalities and abnormal reproductive hormone levels. 

Written informed consent was obtained from each patient before initiation of treatment. All patients were advised to avoid any changes in their physical activity and nutrition and not to undergo any new pharmacotherapy during the study. Patients were divided into four groups considering the similarity of compound variables and randomization in order to minimize the effects of confounding factors through the central method. Patients were treated for three months with intramuscular (i.m.) HCG (Choriomon, Institute Biochemique, Lugano), subcutaneous (s.c.) HMG (Merional, Institute Biochemique, Lugano) and s.c. rhFSH (Gonal-F, Merck Serono, Aubonne, Switzerland) two weeks after varicocelectomy. 

Patients in group A were treated with HCG 5000 IU weekly, patients in group B were treated with HMG 75 IU three times a week, patients in group C were treated with rhFSH 75 IU three times a week and patients in group D received no medical treatment after varicocelectomy which was done by a single surgeon with the inguinal procedure without microsurgery. 

Semen analysis was taken before initiation of treatment and further control examinations were performed 8 to 10 weeks after the completion of each period of treatment. All examinations included recent medical histories of the patient and female partner, detection of adverse events and side effects, physical evaluation and semen analysis. Pregnancies in female partners were recorded a further three months after the last control examination. Primary efficacy end points were improvements of sperm parameters. Pregnancies were considered only as secondary efficacy end points.

Semen was collected after a minimum of three days of abstinence. The spermograms of all the patients reported sperm counts, general motility and morphology before and after the treatment. The specimens were evaluated according to the standard procedures recommended by the World Health Organization (WHO) using the SQALLC-P sperm analysis system. Considering the WHO (1999) classifications, an ejaculate volume >2.0 mL, a sperm concentration >20×10^6^⁄mL, a total sperm count >40×10^6^, >50% sperm motility and >30% normal sperm morphology were regarded as normal.

**Statistical analysis**

All analyses were performed using the statistical software SPSS for Windows version 11.5. Continuous variables were expressed as means (standard deviation) or medians (range). Comparison of the effects of various treatments on patients after varicocelectomy was performed by chi-square test, and comparison of the effect of each treatment before and after it was conducted by paired t-test. 

A two-way analysis of variance (ANOVA) was used in order to take into account any effects resulting from the different treatment centers. The significance of the within-group differences between the values obtained in the screenings was tested by the Wilcoxon signed ranks test. P<0.05 were considered statistically significant.

## Results

**Figure 1:**
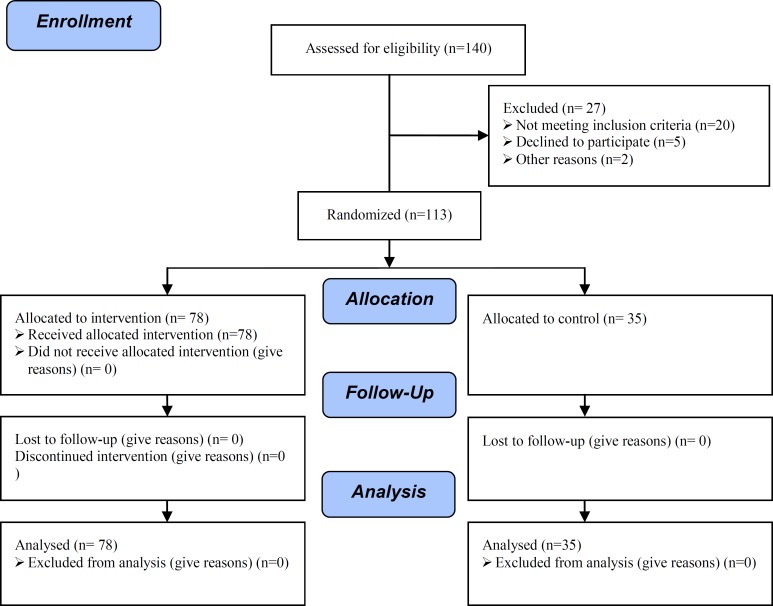
Consort flow chart of RCT

The study was completed from December 2008-2009, during which time 120 men with unilateral left varicocele were recruited. A number of patients from each group did not complete the study. Five men were excluded by their own wishes before starting therapy due to their desires for rapid responses to treatment and intolerance of long-term therapies. Two subjects discontinued treatment for various reasons. Therefore, the study population was comprised of 113 patients (Figure 1). 

Thirty-five subjects did not receive any treatment following varicocelectomy, HCG was administered to 25, HMG to 21 and rhFSH to 32 patients. Parameters of all randomized subjects obtained at the various assessments were analyzed, if available. No statistically significant differences were observed in any of the demographic and basic characteristics among the four groups, such as age, duration of marriage and duration of infertility (Table I), suggesting that they were adequately randomized. 

Subjects in the HCG group had a mean age of 32.4±5.3 years (range 22-45 years), 32.6±6.2 years (range 27-49 years) in the HMG group, 32.28±6.6 years (range 25-42 years) in the rhFSH group and 31.3±5.09 years (range 24-41 years) in the control group. At baseline, no relevant differences with respect to height, weight, body mass index, heart rate, diastolic and systolic blood pressure, and andrological history existed between groups. There were no significant changes in weight, blood pressure, pulse rate or urinalysis during treatment. The median abstinence time calculated over all samples was three to four days, respectively. A difference of approximately 1 mL existed in mean testicular volume among the treatment groups. The testicular volume at the beginning of therapy was 3.38±1.85 in the HCG group, 2.96±1.08 in the HMG group, 3.48±1.61 in the rhFSH group and 3.59±1.80 in the control group. After completion of the treatment course, no significant changes were observed in the results of the semen volume. 

Semen analysis was completed before and after treatment with 5000 IU/week HCG, 75 IU HMG 3 times a week, 75 IU rhFSH three times a week and after varicocelectomy without receiving any drug (Table II, III and IV). Varicocelectomy alone was associated with a slight increase in sperm morphology (p=0.038). After treatment with HCG, no significant differences compared with the pre-treatment sperm characteristics were observed (p=0.803, 0.582) except for the morphology (p=0.007). After treatment with HMG, sperm motility and morphology progressively improved. 

All of the investigated conventional semen parameters increased significantly after treatment with rhFSH compared to baseline values and at the end of the treatment, sperm concentrations of 20×10^6^/mL or higher as the lower limit of the normal range were reached by 17 subjects (53%). The overall mean percentage of morphologically normal sperm cells became 79% and 59% of the spermatozoa were motile. Twenty out of 32 patients successfully induced pregnancies (62.5%). The pregnancy rate following treatment with HCG was reported to be 32% (8 from 25) as compared to 57% (12 from 21) in the HMG group. 

The pregnancies achieved in the treated groups were spontaneous pregnancies. Of the 113 patients who completed the study, 73 could be evaluated for the induction of pregnancies in their partners. Throughout treatment, all couples reported regular intercourse ranging from two to four times per week. Partners of five patients receiving no drug had pregnancies induced with IVF and 10 pregnancies in the placebo group were achieved by microinjection. 

Following the 6 month observation period after treatment, further pregnancies occurred spontaneously or with the aid of ART. The treatments were well-tolerated in all groups and no cases were terminated because of side-effects. No significant differences for the global incidence of adverse events were noted. The following adverse events were reported: mild headache, nausea and mild pain at the injection site. 

**Table I:** Demographic characteristics of the patients enrolled and randomized to treatment with 5000 IU/week HCG, 75 IU HMG 3 times a week, 75 IU Gonal-F three times a week and no drug treatment after varicocelectomy (values are means±SD).

**Characteristics**	**HCG group**	**HMG group**	**Gonal-F group**	**Control group**	**p-value**
Number of patients (%)	25 (22.12)	21 (18.54)	32 (28.31)	35 (30.97)	
Patient`s age (year)	32.04 (5.34)	32.62 (6.22)	32.28 (6.06)	31.03 (5.09)	0.807
Duration of marriage (year)	4.45 (2.56)	3.94 (2.40)	4.67 (2.13)	4.06 (2.39)	0.670
Duration of infertility (year)	2.36 (1.20)	2.02 (1.08)	2.50 (1.17)	2.22 (1.28)	0.767
Sexual abstinence time (days)	3.56 (0.2)	3.46 (0.2)	3.26 (0.2 )	3.36 (0.2)	NS
Ejaculate volume (mL) before treatment	3.38 (1.85)	2.96 (1.08)	3.48 (1.61)	3.59 (1.80)	NS
Ejaculate volume (mL) after treatment	2.86 (1.63)	3.02 (1.42)	2.93 (1.54)	3.41 (1.52)	NS

NS: not significant.

Data are presented as number (%).

**Table II:** Sperm concentration before and after treatment with 5000 IU/week HCG, 75 IU HMG 3 times a week, 75 IU Gonal-F three times a week or after varicocelectomy without receiving any drug

**Characteristics**	**HCG group**	**HMG group**	**Gonal-F group**	**Control group**
Sperm concentration >20×10^6^⁄mL before treatment	12 (48.00)	7 (33.33)	10 (31.25)	14 (40.00)
Sperm concentration >20×10^6^⁄mL after treatment	11 (44.00)	10 (47.62)	17 (53.13)	20 (57.14)
Sperm concentration <20×10^6^⁄mL before treatment	13 (52.00)	14 (66.67)	22 (68.76)	21 (60.00)
Sperm concentration <20×10^6^⁄mL after treatment	14 (56.00)	11 (52.38)	15 (46.88)	15 (42.86)
p-value	0.803	0.130	0.027	0.157

P-value <0.05: significant.

Data are presented as number (%).

**Table III:** Sperm motility before and after treatment with 5000 IU/week HCG, 75 IU HMG 3 times a week, 75 IU Gonal-F three times a week or after varicocelectomy without receiving any drug.

**Characteristics**	**HCG group**	**HMG group**	**Gonal-F group**	**Control group**
Sperm motility >50% before treatment	9 (36.00)	6 (28.57)	15 (46.88)	16 (45.71)
Sperm motility >50% after treatment	11 (44.00)	10 (47.62)	19 (59.38)	18 (51.43)
Sperm motility <50% before treatment	16 (64.00)	15 (71.43)	17 (53.13)	19 (54.28)
Sperm motility <50% after treatment	14 (56.00)	11 (52.39)	13 (40.63)	17 (48.57)
p-value	0.582	0.023	0.027	0.358

P-value <0.05: significant.

Data are presented as number (%).

**Table IV:** Sperm morphology before and after treatment with 5000 IU/week HCG, 75 IU HMG 3 times a week, 75 IU Gonal-F three times a week or after varicocelectomy without receiving any drug

**Characteristics**	**HCG group**	**HMG group**	**Gonal-F group**	**Control group**
Normal sperm morphology >30% before treatment	14 (56.00)	12 (57.14)	20 (62.50)	23 (65.72)
Normal sperm morphology >30% after treatment	20 (80.00)	19 (90.47)	25 (78.14)	30 (85.71)
Normal sperm morphology <30% before treatment	11 (44.00)	9 (42.86)	12 (37.50)	12 (34.29)
Normal sperm morphology <30% after treatment	5 (20.00)	2 (9.52)	7 (21.88)	5 (14.29)
p-value	0.007	0.014	0.015	0.038

P-value < 0.05: significant.

Data are presented as number (%).

## Discussion

In this study, we compared, for the first time, the safety and efficacy of rhFSH, HCG and HMG treatments on sperm parameters and fertility following varicocelectomy. Although the most common type of male infertility is idiopathic, among physical findings, varicocele is 2-3 times more frequent in men who attend infertility clinics than in men of the general population (31-35). 

A multi-center study from the WHO showed that the frequency of varicocele in infertile couples among different geographical regions varied from 6-47% (36). We assessed infertile men who were referred to us and chose only those for whom infertility was due to varicocele, which is defined as the enlargement of the venous plexus of spermatic tone. Increased testicular temperature due to varicocele affects spermatogenesis. 

The low sperm concentration is attributed to high rates of germ cell apoptosis, while the low motility is caused by either the presence of anti-sperm antibodies or the increased concentration of reactive oxygen species (ROS) (14). Normally, the body contains a minimum amount of ROS, since these chemicals are needed for regulating normal sperm functions such as capacitation, the acrosome reaction, and sperm–oocyte fusion (37). 

An excess of ROS, however, can cause oxidative stress which is now recognized as a major factor in infertility (38-40). Oxidative stress has adverse effects on sperm structure and function, such as membrane lipid alterations, disruption of sperm metabolism, reduction of motility, DNA fragmentation and reduced overall sperm quality (41-43). Investigations have shown that varicocelectomy can reduce functional factors not tested during standard semen analysis, such as seminal oxidative stress, even when seminal parameters do not improve (44, 45). 

The Practice Committee of the American Society for Reproductive Medicine (2006) concluded that varicocelectomy should be offered to the male partner in couples attempting to conceive only when all of the following conditions were present: a palpable varicocele, documented couple infertility, a female partner with normal fertility or potentially correctable infertility, and a male partner with one or more abnormal semen parameters or test results showing abnormal sperm function (3, 46). 

Considering these criteria, we performed varicocelectomy and after 8 to 10 weeks in control group D, a significant increase in sperm morphology was seen (p=0.038) as 85% of patients had normal sperm morphology >30% considering the WHO classification. But other sperm parameters remained unchanged during this period and no spontaneous pregnancies occurred in female partners. 

In a review article, nine studies with considerable differences in the inclusion criteria reported no significant benefit from the surgical repair of varicocele and it became evident that pregnancy rates do not increase significantly after varicocelectomy in men with clinical varicocele and abnormal semen parameters (47-56). A meta-analysis including only randomized, controlled trials and observational studies demonstrated that sperm concentration, total motility and sperm morphology according to WHO standards increased significantly after varicocelectomy (57). 

Also, another analysis suggested that varicocelectomy improves fertility by increasing the likelihood of spontaneous pregnancy in female partners (58). Current evidence supports the idea that varicocele size, sperm counts prior to repair, post-operative total motile sperm count, and pre-surgical testicular volume and FSH concentration predict the results after varicocelectomy (59-63). 

Studies have demonstrated that the mean time for semen improvement and spontaneous pregnancy after surgery is approximately five to seven months, respectively (64, 65). Therefore, we investigated drugs to understand if they can accelerate improvement in spermogram and pregnancy rate. We hypothesized that HCG, HMG and rhFSH reduce ROS and as a result, oxidative stress, thereby decreasing the length of treatment.

Research in monkeys has shown that administration of FSH stimulate spermatogenesis and increase sertoli cell secretion (66, 67). Since gonadotrophin-releasing hormone (GnRH) and gonadotrophins are required for normal testicular function, they were applied in idiopathic male infertility for stimulation of spermatogenesis (68). If pulsatile GnRH is not indicated, HCG is used as the source of luteinizing hormone (LH) bioactivity to stimulate testosterone secretion by Leydig cells, whereas HMG is used as the source of FSH to stimulate the Sertoli cells (69, 70). 

Our results illustrated that after three months of treatment with 5000 IU/week HCG i.m., patients showed a significant improvement in sperm morphology (p=0.007) but no significant change was observed in sperm concentration or motility (p>0.05) as compared with the pre-treatment sperm characteristics. 

However, the pregnancy rate following treatment with HCG was reported to be 32% (8 from 25), which is a secondary efficacy endpoint. Similar results have been reported by previous studies such as one case in which 504 subfertile men who underwent surgical correction of varicocele were followed for at least one year. It was shown that empiric use of post-operative HCG therapy improves results significantly (56% improved semen quality and 44% pregnancy rate) (25).

In another investigation, 128 men with proven infertility were treated with HCG. Excellent results were obtained after HCG treatment in the infertile men who underwent varicocelectomy but who did not respond to the operation, good results in the idiopathic oligospermic men with sperm density less than 20 million and indeterminate results in patients with sperm values more than 20 million (26). Administration of HCG to patients who undergo varicocelectomy, but who have persistent subtle Leydig cell dysfunction disclosed by luteinizing hormone releasing hormone (LHRH) assessment, was recommended to stimulate the intratesticular testosterone production (27). 

Additionally, our study revealed that 75 IU HMG s.c. 3 times a week for three months is effective in improving sperm motility (p=0.023) and morphology (p=0.014) and causes a significant rise in pregnancy: 57% (12 out of 21 cases). These results are concordant with those of previous studies. In one investigation, 60 patients with left varicocele were randomized into three groups of 20: group A was treated with menotropin beginning at diagnosis and continuing for three months; group B was treated with menotropin three months after surgical treatment; and group C was treated only with varicocelectomy. 

At three months following surgical treatment, sperm parameters were significantly improved only in group A (p<0.05). After six months, a similar improvement occurred even in group B. After twelve months, significant improvements were recorded in groups A and B; the values were significantly higher than in group C (p<0.05). After six months, the pregnancy rate was 42%, 25% and 22.2% and after twelve months, it was 47%, 45% and 27.7% in the three groups, respectively. These data showed that the association between varicocelectomy and early use of menotropin seems to improve testis functional rehabilitation (28). 

Recently, a recombinant human follicle-stimulating hormone (rhFSH; follitropin alpha) has become available (71, 72). As only a mammalian cell can glycosylate the FSH protein correctly, rhFSH is produced in genetically-engineered Chinese hamster ovary cells in which the genes encoding the alpha and beta chains of human FSH are present to ensure full biological activity (71, 73). rhFSH is manufactured by recombinant DNA technology (74). Studies have demonstrated that rhFSH is significantly more effective than urinary FSH; it has greater purity, higher specific activity, more consistent composition and theoretically unlimited supply. (75-77). 

Two rhFSH preparations are in current clinical use, rhFSH and Puregon (78). Gonal-F is purified from cell culture supernatant by ultra-filtration followed by five chromatographic stages including reversed-phase high performance liquid chromatography with an immunoaffinity procedure as the principal purification step (73). In our evaluations, 75 IU rhFSH s.c. three times a week for three months caused significant increases in all of the investigated conventional semen parameters (p=0.027, 0.027, 0.015). 

It is important that among the drugs investigated in this study, only rhFSH can improve sperm concentrations to >20×10^6^/mL during treatment considering WHO classification (p=0.027). After completion of the treatment course, the pregnancy rate was 62.5% (20 of 32), which is remarkable with this short-term treatment. The efficacy of rhFSH is thought to be due to improved appearance of the sperm head subcellular organelles as visualized by electron microscopy (79, 80). 

A randomized, double-blind, placebo-controlled, clinical trial showed that administration of 150 IU rhFSH for 12 weeks did not lead to an improvement of conventional, biochemical or electron microscopy sperm parameters, but did increase testicular volumes and result in higher sperm DNA condensation in the treated group. These data indicate that FSH is exerting an effect on spermatogenesis and sperm maturation (81).

In a prospective study, 20 boys (age range: 15-20 yrs), affected by left idiopathic varicocele, were treated with high purified urinary FSH s.c. three times a week for three months. At the end of the study, a statistically significant increase in sperm density and a decrease of atypical forms was observed. In this study, FSH treatment in young people with varicocele led to a statistically significant increase in the seminal fluid parameters and characterizes a group of patients who may have better prognostic outcomes regarding tubular gonadal function and thus improved fertility potential in adult life (29). 

A prospective multi-center study concluded that rhFSH is effective in inducing testis growth, spermatogenesis and fertility. The efficacy and safety of this treatment seems comparable with urinary FSH, suggesting similar biological efficacy (82). In another investigation, 183 patients affected with idiopathic left varicocele were surgically treated and divided into three subsets according to sperm count: group A: <10×10^6^⁄mL, group B: 10-20×10^6^⁄mL, and group C: >20×10^6^⁄mL. 

Six months after surgery, 115 patients were treated for 3 months with pure hFSH (75 IU i.m. every other day), while 68 patients treated with placebo served as a control group. After therapy, a significant improvement in sperm parameters was measured in group A including sperm count, motility, morphology and cervical mucus penetration test (CMPT). In group B, sperm motility, viability, CMPT and DNA integrity showed significant improvements, while in group C only a significant improvement of CMPT was observed. Investigators concluded that FSH treatment in patients after varicocelectomy could improve spermatogenesis, particularly in those who demonstrated more compromised sperm quality prior to surgery. 

On the contrary, no significant differences in sperm patterns were recorded in the control group before and after placebo treatment (30). One study highlighted a positive role for FSH therapy in infertile males before intra cytoplasmic sperm injection (ICSI). Treatment with FSH was correlated with increased pregnancy rates probably by improvement of sperm structure which could influence the quality of embryo implantation an development (83). Divergent rates present in different studies might be due to variations in dosages and treatment periods. It seems that the beneficial effects of drugs are manifest in some special groups. Assessment of such an issue by dividing patients into sub-groups should be considered in future studies.

After completion of our treatment course, no significant changes were observed with regard semen volume. We did not compare testis volume and hormone blood levels before and after treatment in our study. The effect of varicocele grade in treatment can be evaluated in future investigations. Regarding the results obtained in this study, as well as those found by other investigators, treatments were well-tolerated in all groups and no significant differences for the global incidence of side-effects were noted.

Although moderate side-effects have been reported in other studies such as acne (52% of patients), gynecomastia (10%) (84), migraine crises (85), and local reactions to injections (30%) (86), we report only mild headache, nausea and mild pain at the injection site, which could be decreased by using less solvent and by administering the product slowly. 

rhFSH was very attractive to patients because it can be self-administered s.c. Furthermore, there was no evidence of allergy or anti-FSH antibody production. These findings confirm the safety of rhFSH as demonstrated in clinical trials in women (87). In contrast to ovarian hyperstimulation in women, manifestations of FSH overdose were not observed in men. 

## ‍Conclusion

In conclusion, this study confirms that HCG, HMG and rhFSH are effective for improvement sperm quality and restoration of fertility in the majority of infertile men after varicocelectomy. Drugs can reduce the induction times for spermatogenesis and fertility, in comparison with varicocelectomy alone, and are well-tolerated. It also can be concluded from the results of the present study that rhFSH is more effective than other drugs at stimulating testis function and caused more pregnancies in a shorter time period than varicocelectomy alone. 

## Footnote

***Registretion ID in IRCT: IRCT201112248509N1.***
